# SWAP-70 promotes glioblastoma cellular migration and invasion by regulating the expression of CD44s

**DOI:** 10.1186/s12935-019-1035-3

**Published:** 2019-11-21

**Authors:** Lin Shi, Huize Liu, Yifeng Wang, Yulong Chong, Jie Wang, Guanzheng Liu, Xu Zhang, Xiangyu Chen, Huan Li, Mingshan Niu, Jun Liang, Rutong Yu, Xuejiao Liu

**Affiliations:** 1 Institute of Nervous System Diseases, Affiliated Hospital of Xuzhou Medical University, Xuzhou Medical University, Xuzhou, Jiangsu China; 2 Department of Neurosurgery, Affiliated Hospital of Xuzhou Medical University, Xuzhou, Jiangsu China; 3 Blood Diseases Institute, Xuzhou Medical University, Xuzhou, Jiangsu China; 4 Nanjing Durm Tower Hospital Group, Suqian City People’s Hospital, Suqian, Jiangsu China

**Keywords:** Glioblastoma, SWAP-70, CD44s, Migration, Invasion

## Abstract

**Background:**

Switch-associated protein 70 (SWAP-70) is a guanine nucleotide exchange factor that is involved in cytoskeletal rearrangement and regulation of migration and invasion of malignant tumors. However, the mechanism by which SWAP-70 regulates the migration and invasion of glioblastoma (GB) cells has not been fully elucidated.

**Methods:**

This study used an online database to analyze the relationship between SWAP-70 expression and prognosis in GB patients. The in vitro wound healing assay and transwell invasion assay were used to determine the role of SWAP-70 in GB cell migration and invasion as well as the underlying mechanism.

**Results:**

We found that patients with high SWAP-70 expression in the GB had a poor prognosis. Downregulation of SWAP-70 inhibited GB cell migration and invasion, whereas SWAP-70 overexpression had an opposite effect. Interestingly, SWAP-70 expression was positively correlated with the expression of the standard form of CD44 (CD44s) in GB tissues. Downregulation of SWAP-70 also reduced CD44s protein expression, whereas SWAP-70 overexpression enhanced CD44s protein expression. However, downregulation of SWAP-70 expression did not affect the mRNA expression of CD44s. Reversal experiments showed that overexpressing CD44s in cell lines with downregulated SWAP-70 partially abolished the inhibitory effects of downregulated SWAP-70 on GB cell migration and invasion.

**Conclusions:**

These results suggest that SWAP-70 may promote GB cell migration and invasion by regulating the expression of CD44s. SWAP-70 may serve as a new biomarker and a potential therapeutic target for GB.

## Background

Glioblastoma (GB) is the most common and malignant type of brain tumor in adults. GB is a highly invasive tumor and its mortality is among the highest of all malignancies in humans [1, 2]. Although comprehensive therapeutic regimens, such as a combination of surgery with radiotherapy and chemotherapy, are used to treat GB in clinical practice, the prognosis of patients is still poor, with a median survival of only 12–15 months and a 5-year overall survival (OS) of less than 6% [3, 4]. The invasive growth of GB represents one of the key challenges for complete resection of such tumors. Hence, there is an urgent need to better understand the molecular mechanisms underlying GB cellular migration and invasion in order to identify potential targets for GB treatment.

Cellular invasion is a complex cellular process that includes cell-to-cell and cell-to-extracellular matrix (ECM) interactions, as well as cellular migration and degradation of ECM enzymes [5–7]. The molecular mechanisms underlying the high migration and invasion of GB have not been fully elucidated. However, migration and invasion must involve a balance between the external environment and intracellular responses, the processes of which dynamically regulate actin filaments, microtubules, and intermediate filaments [8, 9]. Cellular movement is usually driven by the dynamic reorganization of the actin cytoskeleton, which projects to the anterior part of the cell and contracts in the posterior part of the cell [8]. Previous studies have shown that filaments that present inside pseudopods and membranous folds are associated with and are regulated by switch-associated protein 70 (SWAP-70) [10, 11]. SWAP-70 overexpression alters the actin arrangement and morphology of lamellipodia [12]. It has been shown that deletion of SWAP-70 in mouse embryonic fibroblasts results in impaired membrane wrinkles, suggesting that SWAP-70 plays a crucial role in the formation of the membranous cellular folds [13, 14].

SWAP-70 is a phosphatidylinositol triphosphate (PIP3)-binding protein, belonging to the family of guanine-nucleotide exchange factors (GEFs) [15]. SWAP-70 regulates cytoskeletal rearrangement through PIP3-mediated signaling and is expressed in a variety of tissues and cells [9, 16]. SWAP-70 also plays an important role in the oncogenesis of GB, prostate cancer, and other malignant tumors [17–19]. SWAP-70 expression in malignant tumors is higher than that in normal tissues, indicating that it may be closely related to oncogenesis in vivo. Although studies have reported that SWAP-70 regulates cellular movement and tumor-cell migration and invasion, the underlying mechanisms have not been extensively studied. It has only been reported that SWAP-70 is involved in phosphoinositide-3-kinase (PI3K) downstream signaling and activation of Rac, which subsequently regulates cytoskeletal changes that affect membranous cellular fold formation, thereby regulating tumor cell migration and invasion [16, 17]. SWAP-70 is a GEF that is involved in cytoskeletal rearrangement; however, it is unclear if SWAP-70 regulates GB cellular migration and invasion through other mechanisms.

The present study investigated the roles and underlying mechanisms of SWAP-70 in GB cellular migration and invasion. We found that high SWAP-70 expression in high-grade glioma tissues was associated with a poor prognosis of glioma patients. We also found that SWAP-70 regulated the expression of the standard form of CD44 (CD44s) to promote GB cellular migration and invasion. Collectively, our findings provide an experimental basis for the applicability of SWAP-70 as a potential therapeutic target for GB.

## Materials and methods

### Patient tissue samples

A total of 62 patient tissue samples including 15 cases of non-tumorous brain tissues and 47 cases of gliomas tissues (Grade II: 15, Grade III: 18, Grade IV: 14) were used in this study. All the specimens were obtained from the Affiliated Hospital of Xuzhou Medical University (Xuzhou, China). Glioma patients were histologically diagnosed according to World Health Organization (WHO) criteria. None of the patients received any therapies before sample collection, such as radiation, immunotherapy or chemotherapy. Written informed consent was obtained from all of the patients, and this study was approved by the Research Ethics Committee of the Affiliated Hospital of Xuzhou Medical University. All the tissue samples were frozen in liquid nitrogen.

### SWAP-70 expression and survival in patients with glioma

Total protein extracts from non-tumorous brain tissues and glioma tissues (WHO II-IV) were subjected to Western blot analysis as described previously [20]. The expression patterns of SWAP-70 and CD44s were detected using specific antibodies and β-actin was used as the loading control. Kaplan–Meier analysis was performed online with TCGA and GEO databases, and truncation values of the high expression group and the low expression group were determined by automatic scanning. The Kaplan scanner is used to determine the optimal cutoff for gene expression levels.

### Cell lines and antibodies

Cell lines 293T, U251 and U87 were purchased from the Shanghai Cell Bank, Chinese Academy of Sciences for this study. These cell lines were cultured in DMEM supplemented with 10% FBS at 37 °C in a humidified incubator with 5% CO_2_. Antibodies against SWAP-70 (ab89532) and CD44s (ab51037) were obtained from Abcam. Antibodies specific for β-actin (A1978) and Flag (F7425) were purchased from Sigma.

### Lentiviral vector construction

For SWAP-70 silencing, two short hairpin RNA (shRNA) duplexes were designed to target human SWAP-70 gene. The shRNA oligomers were annealed and then subcloned into the pLB plasmid by the HpaI and XhoI cloning site. For overexpression of SWAP-70, the cDNA encoding human SWAP-70 gene was inserted into the pWPXLd-puro lentiviral vector using BamHI and MluI sites. For silencing of CD44s, a pair of shRNA sequences were synthesized and incubated in annealing buffer. Annealed shRNA oligomers were inserted into pLVshRNA-EGFP plasmid digested with BamHI and EcoRI. The CD44s gene fragment was obtained by PCR amplification with the unique restriction sites EcoRI and NotI. After amplification, the PCR product was inserted into the lentiviral vector pLV-EGFP-N. The primers for CD44s overexpression contained a Flag tag. The primers used in this study were shown in Table 1.

**Table 1:** Primer sequences for amplification of SWAP-70 or CD44s using in this study

Primer	Sequences (5′–3′)
sh-SWAP-70 #1 sense	TGCTGGAAGACATGTACCTATTCAAGAGATAGG TACATGTCTTCCAGCTTTTTC
sh-SWAP-70 #1 antisense	TCGAGAAAAAAGCTGGAAGACATGTACCTATCT CTTTGAATAGGTACAGTTCCAGCA
sh-SWAP-70 #2 sense	TGCCCATCATGAAGGATTAATTCAAGAGATTAA TCCTTCATGATGGGTTTTTTC
sh-SWAP-70 #2 antisense	TCGAGAAAAAAGCCCATCATGAAGGATTAATCT CTTGAATTAATCCTTCATGATGGGCA
sh-CD44s sense	ATCGTATGACACATATTGCTTCTTCAAGAGAGA AGCAATATGTGTCATACTTTTTTG
sh-CD44s antisense	TTCAAAAAAGTATGACACATATTGCTTCTCTCTT GAAGAAGCAATATGTGTGTCATAC
pWPXLd-SWAP-70 sense	CGGGATCCATGGGGAGCTTGAAGGAGGA
pWPXLd-SWAP-70 antisense	CGACGCGTCCCTCCGTCCTCTTTTTCTCTTT
pLV-EGFP-CD44s sense	CGGAATTCATGGACAAGTTTTGGTGGC
pLV-EGFP-CD44s antisense	TAAGAATGCGGCCGCGCTACTTGTCATCGTCAT CCTTGTAGTCGATCACCCCAATCTTCATGTCCACs

### Establishment of stable cell lines

A lentiviral packaging system was used in this study as previously described [2]. Briefly, lentiviruses were generated by co-transfecting lentiviral vector and two packaging vectors in a 3:2:1 ratio in 293T cells by use of PolyJet™ transfection reagent. Supernatants were collected 48 h after transfection, passed through a 0.45-μm filter membrane, and used directly to infect cells (U87 or U251 cells).

### Cell migration assay

Cell migration was assessed by a scratch wound assay. Briefly, cells were seeded in 6-well plates and grown to confluency. A scratch wound was created using a plastic pipette tip, detached cells were washed away with PBS, and then the remaining cells were incubated in serum-free media. After 24 h and 48 h incubation, five random fields were photographed using an Olympus IX-71 inverted microscope. The number of migrating cells across the wound was counted on these images.

### Cell invasion assay

Cell invasion assay was performed using a transwell system. Diluted Matrigel with cold distilled water was applied to 8 μm pore size polycarbonate membrane filters. Cells in serum-free medium were plated added to the upper chamber at a density of 10,000 cells/well. In the lower chamber, the DMEM media containing 10% FBS was added. Cells were incubated for 36 h and then the non-invading cells were removed with cotton swabs. Cells that invaded to the bottom of the membrane were fixed in 4% methanol for 20 min and stained with a 0.3% crystal violet solution for 30 min. The number of invading cells was analyzed by a microscope.

### RNA extraction and real-time quantitative PCR

Total RNA was extracted from control and SWAP-70-downregulated cells using Trizol (Invitrogen) according to the manufacturer’s protocol. Reverse transcription was performed by Transcriptor First Strand cDNA Synthesis Kit (Roche). qPCR was performed as our previous study protocol [21]. The following gene-specific primers were used according to previous published study: SWAP-70: 5′-TCCACCATCCATCTGTTGAA-3′ (forward) and 5′-GCGTTTCTTTTCCTCGTCTG-3′ (reverse); CD44s: 5′-TCCAACACCTCCCAGTATGACA-3′ (forward) and 5′-GGCAGG TCTGTGACTGATGTACA-3′ (reverse); GAPDH: 5′-TCAGTGGTGGACCTGACCTG-3′ (forward) and 5′-TGCTGTAGCCAAATTCGT TG-3′ (reverse). GAPDH mRNA levels were used for normalization.

### Statistical analysis

Each experiment was performed at least three times. Results are expressed as the mean ± SEM. The statistical analysis was performed by using GraphPad Prism 6.0 software. Differences between control and experimental groups were assessed by Student’s t-test or One-way ANOVA. *P *< 0.05 were considered to be statistically significant.

## Results

### High SWAP-70 expression in high-grade glioma tissues leads to poor prognoses in glioma patients

**Fig. 1:**
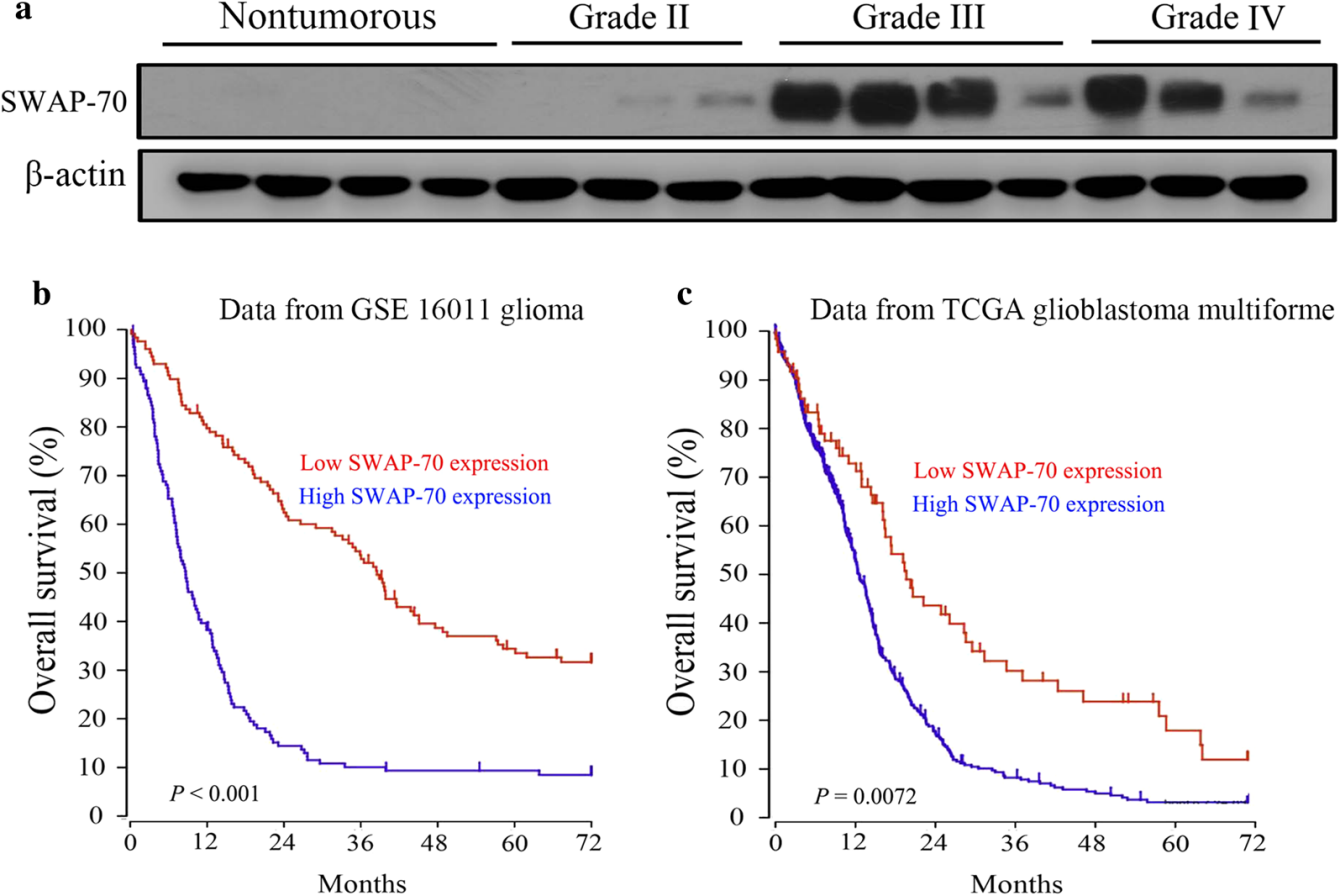
High expression of SWAP-70 correlates with poor outcomes in glioblastoma patients. **a** SWAP-70 expression in non-tumor brain tissues and glioma tissues measured by Western blotting. **b** Kaplan–Meier survival analysis of glioma patients using the Gene Expression Omnibus dataset. Among the 273 glioma cases, 144 tissues had high SWAP-70 expression. **c** Overall survival analysis of glioblastoma patients using The Cancer Genome Atlas glioblastoma dataset

To study the role of SWAP-70 in the pathogenesis of glioma, Western blot analysis was used to measure the SWAP-70 protein expression in glioma tissues and non-tumor brain tissues. As shown in Fig. 1a, SWAP-70 was highly expressed in the high-grade glioma tissues compared to normal brain tissues. Further analysis of the relationship between SWAP-70 expression and the prognosis of glioma patients using online TCGA and GEO databases showed that patients with high SWAP-70 expression in glioma tissues had a poorer prognosis than those with low expression (Fig. 1b, c). High SWAP-70 expression in the high grade glioma tissues was associated with a poor prognosis in high-grade glioma patients, suggesting that high SWAP-70 expression is a poor prognostic marker in the high-grade glioma.

### Downregulation of SWAP-70 inhibits GB cellular migration and invasion

**Fig. 2:**
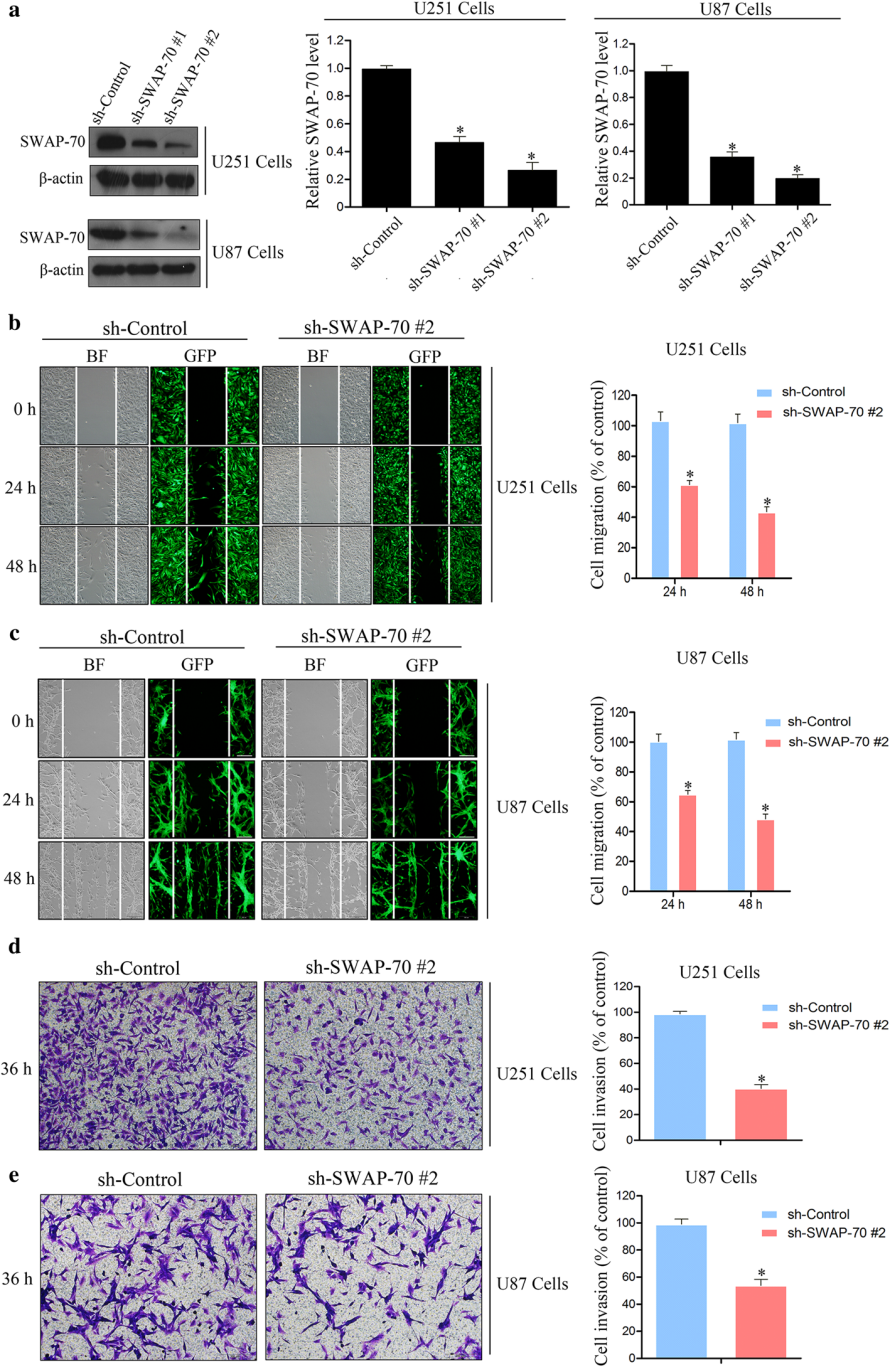
Downregulation of SWAP-70 inhibits glioblastoma cellular migration and invasion. **a** Downregulation efficiency of SWAP-70 in U251 and U87 cells validated by Western blotting. **b**, **c** Migratory abilities of U251 and U87 cells after downregulating SWAP-70 expression detected with the in vitro scratch assay. The number of migrated cells was counted. **d**, **e** Invasive abilities of U251 and U87 cells after downregulating SWAP-70 expression assessed with the transwell invasion assay. The number of invading cells was counted. All data are expressed as the mean ± SEM (n = 3 per group; *P < 0.05)

To investigate the role of SWAP-70 in GB cellular migration and invasion, SWAP-70 expression was downregulated in GB cells using *SWAP*-*70* gene sequence-specific short hairpin RNA (shRNA). The effects of SWAP-70 downregulation on GB cellular migration and invasion were tested using an in vitro scratch assay and transwell invasion assay. First, GB U87 and U251 cell lines with stably downregulated SWAP-70 expression were constructed. Western blotting was used to detect the downregulating efficiency of SWAP-70. As shown in Fig. 2a, the silencing efficiency of shRNA sequence #2 was better than that of any of the other shRNAs, and SWAP-70 protein expression was downregulated by approximately 70–80%. Thus, the shRNA-SWAP-70 #2 cell line was used for the subsequent in vitro scratch and transwell invasion assays.

Results of the in vitro scratch assay showed that the wound healing rate of the control group was faster than that of the SWAP-70 downregulation group at 24 and 48 h after scratching. Statistical analysis showed that after SWAP-70 downregulation, the migration rates of U251 cells decreased by approximately 40% and 57% at 24 and 48 h, respectively, after cell scratching. The migratory rates of U87 cells decreased by approximately 35% and 52% at 24 and 48 h, respectively, after cell scratching (Fig. 2b, c). Similarly, results of the transwell invasion assay showed that after SWAP-70 downregulation, GB cellular invasion was significantly decreased compared to that of the control group. Additionally, compared with those of the control group, the numbers of U251 and U87 cells that penetrated the Matrigel upon SWAP-70 downregulation were reduced by 62% and 48%, respectively (Fig. 2d, e). These results indicated that SWAP-70 downregulation significantly reduced the migratory and invasive abilities of GB cells.

### SWAP-70 overexpression promotes GB cellular migration and invasion

**Fig. 3:**
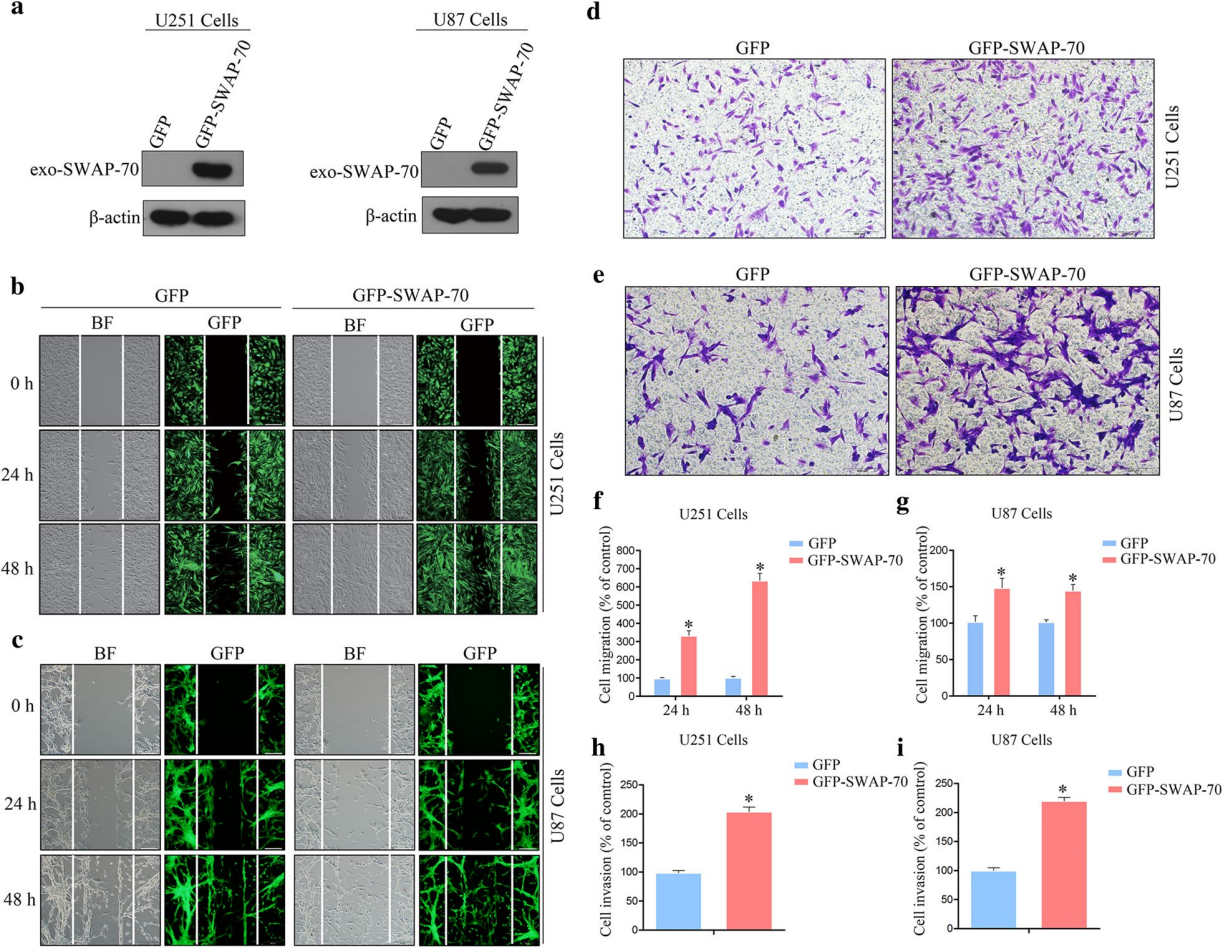
SWAP-70 overexpression promotes glioblastoma cellular migration and invasion. **a** Overexpression efficiency of SWAP-70 in U251 and U87 cells verified by Western blotting. **b**, **c** Migratory abilities of U251 and U87 cells after overexpressing SWAP-70 determined with the in vitro scratch assay. **d**, **e** Invasive abilities of U251 and U87 cells after overexpression of SWAP-70 measured with the transwell invasion assay. **f**–**i** Quantitative analysis of the numbers of invading and migrating cells compared with those of the control group. All data are presented as the mean ± SEM (n = 3 per group; *P < 0.05)

A gain-of-function approach was used in the following experiments to determine the effects of SWAP-70 overexpression on GB cellular migration and invasion. A lentiviral vector system was used to construct U251 and U87 cell lines that stably overexpressed SWAP-70. Western blotting was used to detect the overexpression efficiency of SWAP-70. As shown in Fig. 3a, the significant expression of exogenous SWAP-70 was detected in the SWAP-70 overexpression group compared with that of the control group, indicating successful construction of the U251 and U87 cell lines with SWAP-70 overexpression. The in vitro scratch assay showed that the number of SWAP-70-overexpressing U251 cells migrating to the scratch area was significantly higher than that of the control group, and the migration rates of U251 cells in this experimental group increased by approximately 3.3- and 6.2-fold at 24 h and 48 h, respectively (Fig. 3b, f). Similar results were found in U87 cells, indicating that SWAP-70 overexpression enhanced GB cellular migration (Fig. 3c, g). Similarly, in the transwell invasion assay, the numbers of SWAP-70-overexpressing U251 and U87 cells that penetrated the Matrigel of the transwell chamber increased by 2.1- and 2.3-fold, respectively, compared to those of the control group (Fig. 3d, e, h, i). The above-mentioned results suggest that SWAP-70 overexpression promoted GB cellular migration and invasion.

### SWAP-70 regulates protein expression of CD44s in GB

**Fig. 4:**
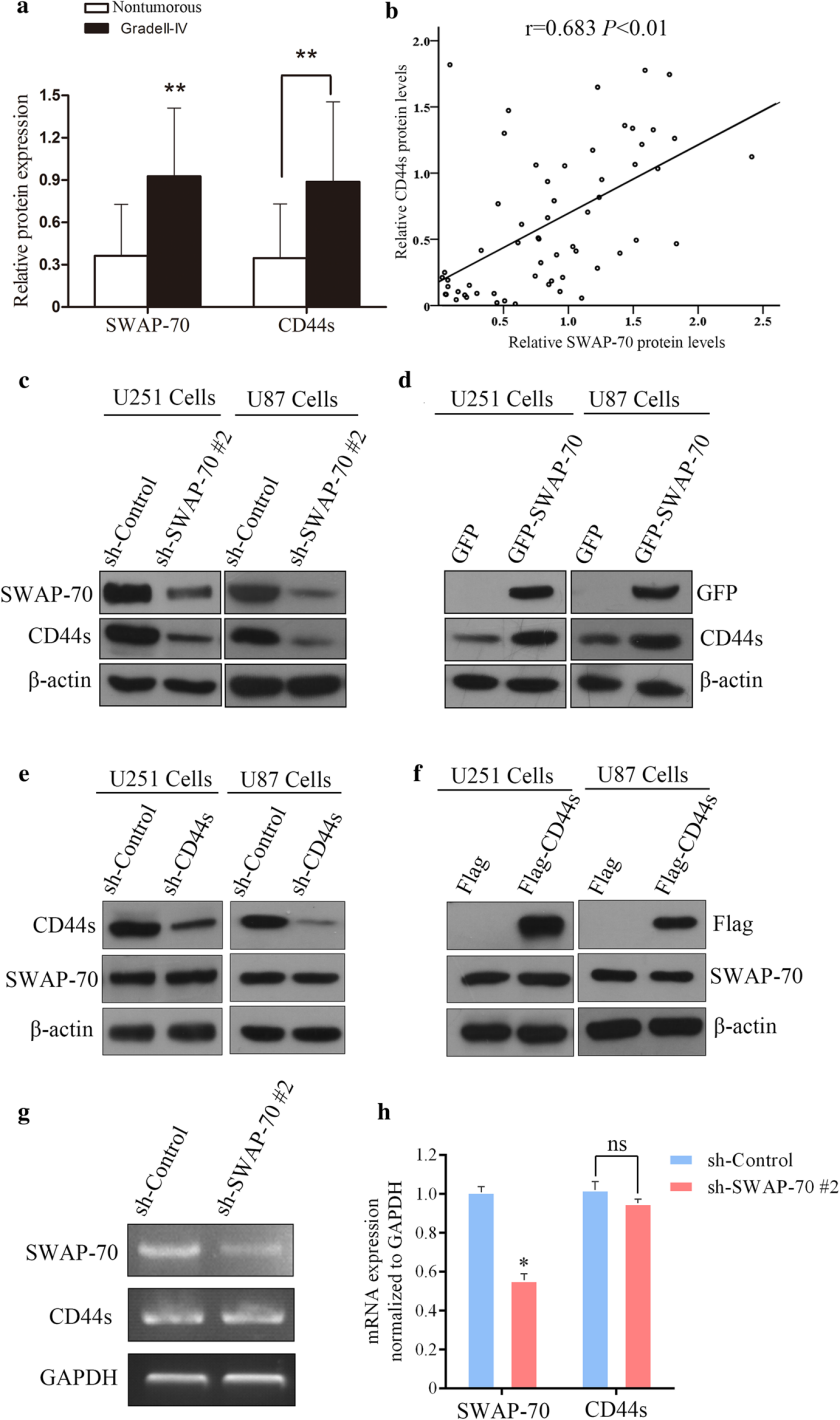
SWAP-70 regulates the expression of CD44s in glioblastoma cells. **a** Correlation analysis of SWAP-70 and CD44s expression in glioblastoma tissues. **b**, **c** Effects of downregulated or overexpressed SWAP-70 on CD44s protein expression analyzed by Western blotting. **d**, **e** Effects of downregulated or overexpressing CD44s on SWAP-70 protein expression assessed by Western blotting. **f** Effects of downregulated SWAP-70 on *CD44s* mRNA expression determined by semi-quantitative PCR. **g** Relative mRNA expression of *CD44s* in cells with stable SWAP-70 downregulation measured by quantitative PCR

Research has shown that binding between CD44s and hyaluronic acid, as well as other adhesion receptors, is a key step in the formation of intracranial inflammatory lesions, in which lymphocytes adhere to the ECM of the brain, penetrate the white matter, and continue to spread [22, 23]. CD44s are key regulators of GB invasive growth and are involved in GB cellular migration and invasion [24–26]. In the present study, SWAP-70 and CD44s were highly expressed in GB tissues. Interestingly, statistical analysis of SWAP-70 and CD44s protein levels showed that SWAP-70 protein expression in GB tissues was significantly and positively correlated with CD44s protein expression in GB tissues with a correlation coefficient of r = 0.683, suggesting that SWAP-70 and CD44s may have a regulatory relationship in the development and progression of GBs (Fig. 4a, b).

Furthermore, cells with downregulated SWAP-70 also had reduced CD44s protein expression, whereas cells overexpressing SWAP-70 demonstrated increased CD44s protein expression (Fig. 4c, d). To determine the upstream and downstream regulation relationship of SWAP-70 and CD44s, we established GB cell lines with downregulated and overexpressing CD44s. Western blotting was used to determine the downregulating and overexpressing efficiencies of CD44s. The changes in CD44s protein expression did not affect SWAP-70 protein expression, suggesting that SWAP-70 is upstream of CD44s and regulates its protein expression (Fig. 4e, f).

To clarify whether SWAP-70 regulates the expression of CD44s at the protein or nucleic acid level, we conducted semi-quantitative PCR and quantitative PCR experiments to determine whether the expression of CD44s is regulated by SWAP-70 at the mRNA level. As shown in Fig. 4g, h, *SWAP*-*70* mRNA expression was significantly reduced after downregulation of SWAP-70; however, downregulation did not affect the mRNA expression of *CD44s*, suggesting that SWAP-70 regulates CD44s protein expression but does not affect its mRNA expression.

### CD44s overexpression reverses the inhibitory effects of downregulated SWAP-70 on GB cellular migration and invasion

**Fig. 5:**
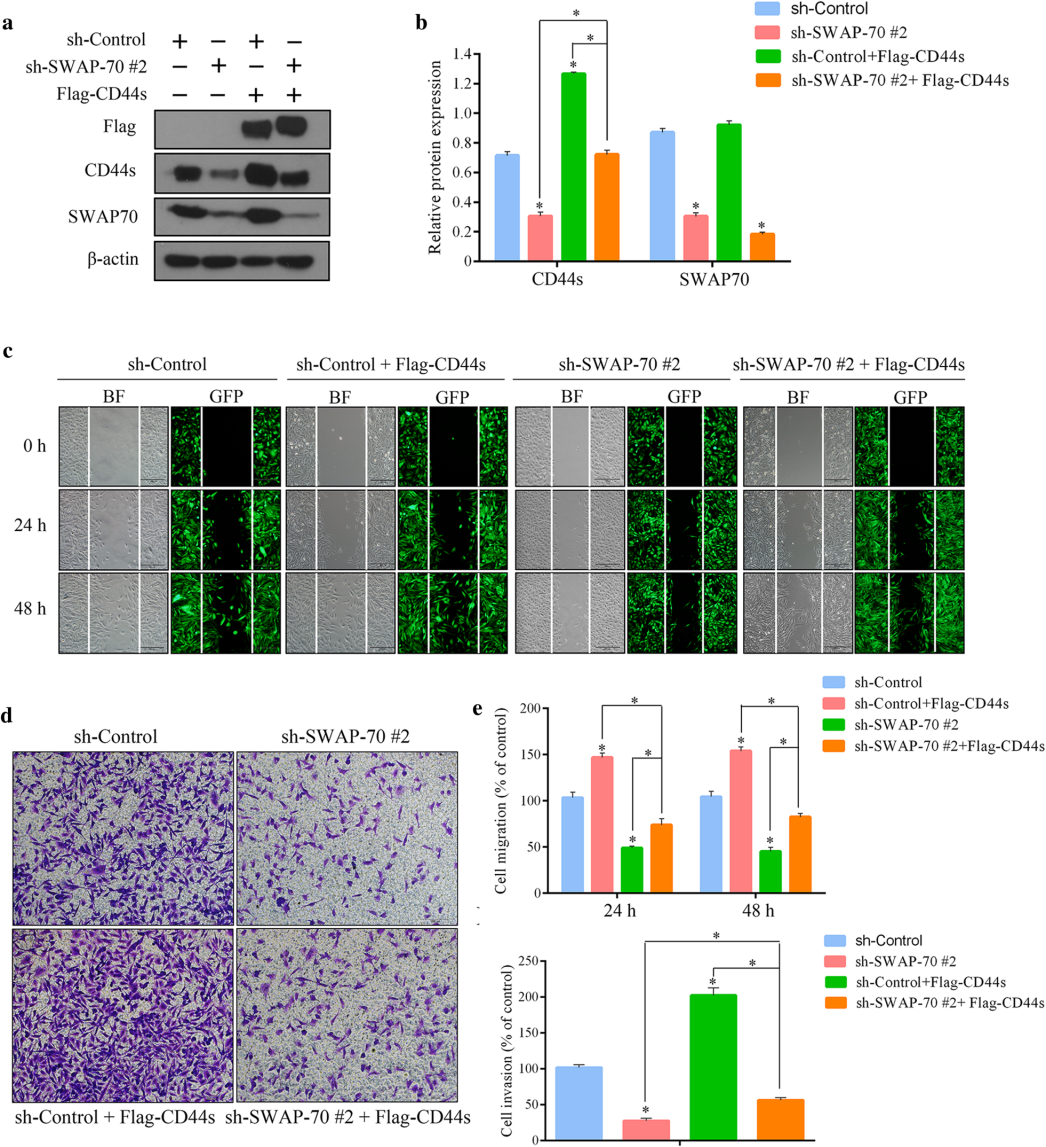
CD44s overexpression partially reverses the inhibitory effect of downregulated SWAP-70 on glioblastoma cellular migration and invasion. **a** Transient transfection of the CD44s-overexpression plasmid in U251 cells with stable SWAP-70 downregulation. Transfection efficiency and protein expression of SWAP-70 and CD44s were assessed by Western blotting. **b** Statistical analysis of SWAP-70 and CD44s protein levels. **c** Effect of CD44s overexpression on the migratory ability of glioblastoma cells after downregulating SWAP-70 determined with the in vitro scratch assay. **d** Effect of CD44s overexpression on the invasive ability of glioblastoma cells after downregulating SWAP-70 analyzed with the transwell invasion assay. **e** Statistical analysis of the numbers of migrating and invading cells. All data are presented as the mean ± SEM (n = 3 per group; *P < 0.05)

To determine if SWAP-70 promoted GB cellular migration and invasion by regulating CD44s, we overexpressed CD44s in U251 cells with stable downregulation of SWAP-70 to test whether the overexpression of CD44s reversed the inhibitory effects of downregulated SWAP-70 on GB cellular migration and invasion. First, Western blotting was used to identify the efficiency of transient transfection of the CD44s overexpression vector. As shown in Fig. 5a, compared with that of the control group, the sh-control cells transfected with CD44s-overexpression plasmids had significantly higher CD44s protein expression. In contrast, cells with downregulated SWAP-70 that were transfected with the CD44s-overexpression plasmid demonstrated less CD44s overexpression, which was a consequence of SWAP-70-mediated downregulation. These results further confirmed that SWAP-70 regulates the protein expression of CD44s (Fig. 5a, b). Subsequently, we performed an in vitro scratch assay and transwell invasion assay (reversal test), and results showed that CD44s overexpression in the cells with downregulated SWAP-70 partially reversed the inhibitory effects of downregulated SWAP-70 on GB cellular migration and invasion (Fig. 5c–e). In summary, our results suggested that SWAP-70 may promote GB cellular migration and invasion by regulating CD44s expression.

## Discussion

The invasive growth of GB makes complete tumor resection challenging and induces the tumor to be prone to recurrence [27, 28]. Elucidating the molecular mechanisms underlying GB cellular migration and invasion, as well as screening for potential therapeutic targets, have received significant attention in the GB research community. The present study showed that SWAP-70 was highly expressed in human high-grade glioma tissues, and high-grade glioma patients with high expression of SWAP-70 in tumor tissues had a poorer prognosis and shorter median survival time compared to those with low SWAP-70 expression, suggesting that SWAP-70 plays an important role in the degree of malignancy of GBs. To verify these findings, we clarified that SWAP-70 downregulation inhibited GB cellular migration and invasion. In contrast, SWAP-70 overexpression promoted GB cellular migration and invasion. Importantly, our results suggest that SWAP-70 in GB may regulate CD44s protein expression to affect GB cellular migration and invasion.

As SWAP-70 is a GEF, it participates in cytoskeletal rearrangement through guanosine triphosphate-guanosine diphosphate conversion and promotes the formation of membrane folds and pseudopods, thereby regulating cellular movement [29, 30]. Recently, many studies have shown that SWAP-70 is involved in the activation of B-cell signaling and may have potential carcinogenic functions in tumorigenesis [18, 19, 31]. Heerema et al. conducted immunohistochemistry on 86 cases of human B-cell neoplasms, and all six cases of nodular lymphocyte-predominant Hodgkin lymphoma had positive SWAP-70 staining [32]. Fukui et al. studied mouse embryo fibroblasts (MEFs) and showed that cells lacking SWAP-70 expression failed to grow on soft agarose, whereas cells overexpressing SWAP-70 were susceptible to cloning. Inoculation of MEFs into nude mice showed that tumor volumes from MEFs lacking SWAP-70 were significantly smaller than those from MEFs expressing exogenous SWAP-70 [33]. These results indicate that the carcinogenesis of MEFs requires the involvement of SWAP-70, suggesting that SWAP-70 may be an oncogene. SWAP-70 is involved in tumor cell migration and invasion in GB [17]; however, the underlying mechanism has not been fully clarified. In the present study, we established GB cell lines with stable SWAP-70 downregulation and SWAP-70 overexpression and used in vitro scratch assays and transwell invasion assays to show that SWAP-70 promoted GB cellular migration and invasion. Interestingly, SWAP-70 expression was positively correlated with the expression of CD44s in GB tissues. Downregulation of SWAP-70 also reduced CD44s protein expression. In contrast, SWAP-70 overexpression enhanced CD44s protein expression. We suspect that there may be a regulatory relationship between the two proteins in GB invasion and metastasis, and that both SWAP-70 and CD44s may be synergistically involved in GB cellular migration and invasion.

CD44 is a transmembrane glycoprotein that is expressed in the plasma membrane. The human *CD44* gene is located on the short arm of chromosome 11 and has 20 highly conserved exons [34]. CD44 molecules are classified into the following two categories based on the types of exons contained in the *CD44* gene: CD44s and the variant isoform of CD44 (CD44v) [35]. The structural difference between CD44s and CD44v is mainly in terms of the extracellular segment. Alternative splicing during transcription of the structural variation region in the extracellular segment results in different protein expression levels [35]. CD44 is involved in intercellular adhesion. CD44 binds to cytoskeletal proteins and participates in cellular movement, as well as intracellular and extracellular signal transduction [36, 37]. Abnormal expression of CD44 significantly enhances tumor cell migration, which is closely related to tumor metastasis [35, 38, 39]. Research has shown that CD44s is the predominant form in human GB, accounting for approximately 83.3% of CD44 types [40]. CD44s plays an important role in GB cellular migration and invasion and is involved in a variety of signaling pathways to promote GB cellular migration and invasion [24, 26]. In addition, the binding of the GEF T-cell lymphoma invasion and metastasis 1 (Tiam 1) to the intracellular domain of CD44v3 promotes breast cancer cellular invasion and migration [41]. The intracellular domain of CD44 is conserved between CD44s and CD44v. SWAP-70, another GEF, may also have a similar mechanism to regulate GB invasion and metastasis. In the present study, our results showed that changes in SWAP-70 expression affected CD44s expression, while CD44s did not affect SWAP-70 expression, suggesting that SWAP-70 may be upstream of CD44s and therefore regulate CD44s protein expression. Further experiments showed that SWAP-70 did not affect the mRNA expression of *CD44s* but regulated the expression of CD44s protein. The dissociation of CD44 is regulated by protein kinase C, calcium ion flux, and small G-proteins of the Ras homologous (Rho) family. The Rho family molecules recruit metalloproteinases to surround CD44, promote CD44 dissociation, and alter the CD44 distribution on the plasma membrane [42]. SWAP-70 regulates cytoskeletal changes through Rac activation. Further studies will be necessary to determine if SWAP-70 affects CD44s expression through Rho family molecules. In addition, we overexpressed CD44s in GB cell lines with downregulated SWAP-70 and showed that CD44s overexpression partially reversed the inhibitory effect of the downregulated SWAP-70 on GB cellular migration and invasion. The above-mentioned results suggest that SWAP-70 is involved in GB cellular migration and invasion via regulation of CD44s expression.

## Conclusion

Taken together, the results of this study demonstrate that high SWAP-70 expression is a biomarker for poor prognosis of GB. SWAP-70 plays an important role in GB cellular migration and invasion. Importantly, SWAP-70 promotes GB cellular migration and invasion by regulating CD44s expression. Thus, SWAP-70 may serve as a novel biomarker and potential target for the treatment of human GB.

## Data Availability

The datasets supporting the conclusions of this article are included within the article.

## Abbreviations

GB: glioblastoma
SWAP-70: switch-associated protein 70
GEF: guanine nucleotide exchange factor
ECM: extracellular matrix

## References

[1] Fabian D, Guillermo Prieto Eibl MDP, Alnahhas I, Sebastian N, Giglio P, Puduvalli V, Gonzalez J, Palmer JD (2019). Treatment of glioblastoma (GB) with the addition of tumor-treating fields (TTF): a review. Cancers.

[2] Liu XJ, Chong YL, Tu YM, Liu N, Yue CL, Qi ZL, Liu HZ, Yao Y, Liu HM, Gao SF (2016). CRM1/XPO1 is associated with clinical outcome in glioma and represents a therapeutic target by perturbing multiple core pathways. J Hematol Oncol.

[3] Lee E, Yong RL, Paddison P, Zhu J (2018). Comparison of glioblastoma (GB) molecular classification methods. Semin Cancer Biol.

[4] Shergalis A, Bankhead A, Luesakul U, Muangsin N, Neamati N (2018). Current challenges and opportunities in treating glioblastoma. Pharmacol Rev.

[5] Friedl P, Alexander S (2011). Cancer invasion and the microenvironment: plasticity and reciprocity. Cell.

[6] Conlon GA, Murray GI (2019). Recent advances in understanding the roles of matrix metalloproteinases in tumour invasion and metastasis. J Pathol.

[7] Brown GT, Murray GI (2015). Current mechanistic insights into the roles of matrix metalloproteinases in tumour invasion and metastasis. J Pathol.

[8] Tang DD, Gerlach BD (2017). The roles and regulation of the actin cytoskeleton, intermediate filaments and microtubules in smooth muscle cell migration. Respir Res.

[9] Zielinski A, Linnartz C, Pleschka C, Dreissen G, Springer R, Merkel R, Hoffmann B (2018). Reorientation dynamics and structural interdependencies of actin, microtubules and intermediate filaments upon cyclic stretch application. Cytoskeleton.

[10] Baranov MV, Revelo NH, Dingjan I, Maraspini R, Ter Beest M, Honigmann A, van den Bogaart G (2016). SWAP70 organizes the actin cytoskeleton and is essential for phagocytosis. Cell Rep.

[11] Ihara S, Oka T, Jessberger R, Fukui Y (2004). Involvement of SWAP-70 in membrane ruffling thorough its F-actin binding domain. Mol Biol Cell.

[12] Hilpela P, Oberbanscheidt P, Hahne P, Hund M, Kalhammer G, Small JV, Bahler M (2003). SWAP-70 identifies a transitional subset of actin filaments in motile cells. Mol Biol Cell.

[13] Fukui Y, Tanaka T, Tachikawa H, Ihara S (2007). SWAP-70 is required for oncogenic transformation by v-Src in mouse embryo fibroblasts. Biochem Biophys Res Commun.

[14] Fukui Y, Morishita K, Ichikawa T, Jessberger R (2014). SWAP-70 contributes to spontaneous transformation of mouse embryo fibroblasts. Mol Biol Cell..

[15] Shinohara M, Terada Y, Iwamatsu A, Shinohara A, Mochizuki N, Higuchi M, Gotoh Y, Ihara S, Nagata S, Itoh H (2002). SWAP-70 is a guanine-nucleotide-exchange factor that mediates signalling of membrane ruffling. Nature.

[16] Chacon-Martinez CA, Jessberger R (2010). Interaction of the cytoskeletal control protein SWAP-70 with Rho GTPases. FEBS J.

[17] Seol HJ, Smith C, Salhia B, Rutka JT (2009). The role of the guanine-nucleotide-exchange factor Swap-70 in the migration and invasiveness of human malignant glioma cells. Tansl Oncol.

[18] Shu CL, Su LC, Chuu CP, Fukui Y (2013). SWAP-70: a new type of oncogene. PLoS ONE.

[19] Chiyomaru T, Tatarano S, Kawakami K, Enokida H, Yoshino H, Nohata N, Fuse M, Seki N, Nakagawa M (2011). SWAP70, actin-binding protein, function as an oncogene targeting tumor-suppressive miR-145 in prostate cancer. Prostate.

[20] Yue CL, Niu MS, Shan QQ, Zhou T, Tu YM, Xie P, Hua L, Yu RT, Liu XJ (2017). High expression of Bruton’s tyrosine kinase (BTK) is required for EGFR-induced NF-kappa B activation and predicts poor prognosis in human glioma. J Exp Clin Cancer Res.

[21] Tu YM, Niu MS, Xie P, Yue CL, Liu N, Qi ZL, Gao SF, Liu HM, Shi Q, Yu RT (2017). Smoothened is a poor prognosis factor and a potential therapeutic target in glioma. Sci Rep.

[22] Tirella A, Kloc-Muniak K, Good L, Ridden J, Ashford M, Puri S, Tirelli N (2019). CD44 targeted delivery of siRNA by using HA-decorated nanotechnologies for KRAS silencing in cancer treatment. Int J Pharm.

[23] Krolikoski M, Monslow J, Pure E (2019). The CD44-HA axis and inflammation in atherosclerosis: a temporal perspective. Matrix Biol.

[24] Mooney KL, Choy W, Sidhu S, Pelargos P, Bui TT, Voth B, Barnette N, Yang I (2016). The role of CD44 in glioblastoma multiforme. J Clin Neurosci.

[25] Vaillant BD, Bhat K, Sulman EP, Balasubramaniyan V, Wang S, Aldape KD, Colman H (2011). CD44 as a prognostic and predictive marker for GBM. J Clin Oncol.

[26] Daniel PM, Filiz G, Mantamadiotis T (2016). Sensitivity of GB cells to cAMP agonist-mediated apoptosis correlates with CD44 expression and agonist resistance with MAPK signaling. Cell Death Dis.

[27] Mughal AA, Zhang L, Fayzullin A, Server A, Li Y, Wu Y, Glass R, Meling T, Langmoen IA, Leergaard TB (2018). Patterns of invasive growth in malignant gliomas—the hippocampus emerges as an invasion-spared brain region. Neoplasia.

[28] Kim EH, Song HS, Yoo SH, Yoon M (2016). Tumor treating fields inhibit glioblastoma cell migration, invasion and angiogenesis. Oncotarget.

[29] Audzevich T, Pearce G, Breucha M, Gunal G, Jessberger R (2013). Control of the STAT6-BCL6 antagonism by SWAP-70 determines IgE production. J Immunol.

[30] Ihara S, Oka T, Fukui Y (2006). Direct binding of SWAP-70 to non-muscle actin is required for membrane ruffling. J Cell Sci.

[31] Pearce G, Angeli V, Randolph GJ, Junt T, von Andrian U, Schnittler HJ, Jessberger R (2006). Signaling protein SWAP-70 is required for efficient B cell homing to lymphoid organs. Nat Immunol.

[32] Heerema AE, Abbey NW, Weinstein M, Herndier BG (2004). Expression of the diffuse B-cell lymphoma family molecule SWAP-70 in human B-cell neoplasms: immunohistochemical study of 86 cases. Appl Immunohistochem Mol Morphol.

[33] Murugan AK, Ihara S, Tokuda E, Uematsu K, Tsuchida N, Fukui Y (2008). SWAP-70 is important for invasive phenotypes of mouse embryo fibroblasts transformed by v-Src. IUBMB Life.

[34] Xu HX, Tian YJ, Yuan X, Wu H, Liu Q, Pestell RG, Wu KM (2015). The role of CD44 in epithelial–mesenchymal transition and cancer development. Oncotargets Ther.

[35] Chen C, Zhao S, Karnad A, Freeman JW (2018). The biology and role of CD44 in cancer progression: therapeutic implications. J Hematol Oncol.

[36] Nam K, Oh S, Lee KM, Yoo SA, Shin I (2015). CD44 regulates cell proliferation, migration, and invasion via modulation of c-Src transcription in human breast cancer cells. Cell Signal.

[37] Sacks JD, Main HG, Muralidhar GG, Elfituri O, Xu HL, Kajdacsy-Balla AA, Barbolina MV (2018). Adhesion and beyond: CD44 in ovarian cancer spheroids. Clin Cancer Res.

[38] Mao MY, Zheng XJ, Jin BH, Zhang FB, Zhu LY, Cui LN (2017). Effects of CD44 and E-cadherin overexpression on the proliferation, adhesion and invasion of ovarian cancer cells. Exp Ther Med.

[39] Ijuin T, Takeuchi Y, Shimono Y, Fukumoto M, Tokuda E, Takenawa T (2016). Regulation of CD44 expression and focal adhesion by Golgi phosphatidylinositol 4-phosphate in breast cancer. Cancer Sci.

[40] Ranuncolo SM, Ladeda V, Specterman S, Varela M, Lastiri J, Morandi A, Matos E, Bal de Kier Joffe E, Puricelli L, Pallotta MG (2002). CD44 expression in human gliomas. J Surg Oncol.

[41] Bourguignon LY, Zhu H, Shao L, Chen YW (2000). CD44 interaction with tiam1 promotes Rac1 signaling and hyaluronic acid-mediated breast tumor cell migration. J Biol Chem.

[42] Okamoto I, Kawano Y, Matsumoto M, Suga M, Kaibuchi K, Ando M, Saya H (1999). Regulated CD44 cleavage under the control of protein kinase C, calcium influx, and the Rho family of small G proteins. J Biol Chem.

